# Therapeutic medical physicist well‐being and employment satisfaction during the COVID‐19 pandemic in California

**DOI:** 10.1002/acm2.70233

**Published:** 2025-09-01

**Authors:** Xiaoyu Liu, Jennifer Zhang, Fagen Xie, Dan Ruan, X. Sharon Qi, Zhilei L. Shen, Varun Sehgal, Amy S. Yu, Steve Goetsch

**Affiliations:** ^1^ Department of Radiation Oncology University of California San Francisco California USA; ^2^ Drexel University College of Medicine Philadelphia Pennsylvania USA; ^3^ Department of Research and Evaluation Kaiser Permanente Southern California California USA; ^4^ Department of Radiation Oncology & Department of Bioengineering University of California, Los Angels Los Angeles California USA; ^5^ Department of Radiation Oncology University of California, Los Angeles Los Angeles California USA; ^6^ Department of Radiation Oncology University of Southern California Los Angeles California USA; ^7^ Department of Radiation Oncology University of California, Irvine Orange County California USA; ^8^ Department of Radiation Oncology Stanford University San Jose California USA; ^9^ San Diego Gamma Knife Center La Jolla California USA

**Keywords:** burnout, correlation coefficient, COVID‐19, job status, medical physicist, stress, workforce

## Abstract

**Purpose:**

This study aims to investigate medical physicist job status during the COVID‐19 pandemic in California and collect community feedback on strategies to alleviate job burnout and stress to improve overall work‐life balance.

**Methods and materials:**

The online survey platform SurveyMonkey was used to generate a 14‐question survey, enabling data collection from a large pool of respondents. The 14 survey questions were designed to assess respondent demographics, measuring eight different variables related to medical physicist job status, and collecting feedback regarding burnout and job stress. The American Association of Physicists in Medicine (AAPM) Southern California Chapter Education Committee emailed out the survey link to all AAPM Southern California and Northern California chapter members, requesting that each person provide a single response. All responses were anonymized, and data were analyzed using both qualitative and quantitative methods.

**Results:**

At the end of a 6‐week collection period (January 5, 2022, to February 18, 2022), a total of 97 responses (28.7%) were collected out of 338 invitations. Our analysis results show that “Burnout Level” is strongly and positively correlated with “Leaving Job” (Correlation Coefficient CC = 0.66, *p* < 0.05), “Work Environment” (CC = 0.54, *p* < 0.05), and “Stress Level” (CC = 0.70, *p* < 0.05); it is moderately and negatively correlated with “Salary Satisfaction” (CC = ‐0.49, *p* < 0.05) and “Job Satisfaction” (CC = ‐0.48, *p* < 0.05). Qualitative results revealed that the seven most common recommendation categories to alleviate job burnout and stress from respondents were: adequate staffing, culture of recognition, salary increase, team building, structure and organization, flexibility of remote working, and physicist involvement in decision‐making.

**Conclusion:**

This study provides an overview of the job status of California‐based medical physicists during the COVID‐19 pandemic, offers valuable insights into managing job‐related stress and burnout. These findings underscore the need for tailored interventions and policy considerations to alleviate burnout, enhance job satisfaction.

## 1 INTRODUCTION

In May 2022, during Mental Health Awareness Month, the U.S. Surgeon General's Advisory spotlighted the profound impact of the COVID‐19 pandemic on healthcare workers. The surge in burnout among healthcare professionals has been a mounting national concern over the past decade, a crisis that began unfolding even before the onset of the COVID‐19 pandemic.^1^ The impact of the pandemic not only dramatically impacted the healthcare system but also exacerbated the group of healthcare workers already grappling with alarming levels of burnout prior to the pandemic. During the first wave of the COVID‐19 pandemic, Couarraze and colleagues^2^ conducted an international study that found healthcare professionals were experiencing the highest levels of stress versus other non‐healthcare workers. Burnout is defined as emotional exhaustion, depersonalization, and a sense of low personal accomplishment that leads to decreased effectiveness at work and in people's quality of life.^3^ While burnout can affect individuals across various professions, healthcare workers appear to be particularly vulnerable due to the nature of their jobs.^3^ A significant amount of research on employee stress and well‐being has shown that job stress has a direct impact on employee satisfaction, commitment, turnover, absenteeism, and performance.^4^ On the individual level, work‐related stress and burnout are also associated with various health consequences and harmful coping strategies, such as smoking, alcohol consumption, and substance abuse.^5^ Previous studies have found that burnout was a significant predictor for both physical health and psychological consequences, such as type 2 diabetes, coronary heart disease, insomnia, and depression.^6^ Notably, burnout extends its reach beyond personal well‐being, as it can have severe professional consequences, including compromised quality of care, diminished patient satisfaction, and an increased likelihood of medical errors.

Given these pressing concerns, it becomes imperative to study the issue of job stress and burnout within the field of radiation oncology, specifically medical physics. Additionally, it is crucial to explore coping mechanisms and potential solutions to address this pressing challenge. Previous studies have identified several practice changes in the field of radiation oncology during the COVID‐19 pandemic, such as increased use of telemedicine, new safety protocols, and treatment delays.^7^, ^8^ Our research initiative aims to shed light on the job status of medical physicists during the COVID‐19 pandemic, focusing on members of The American Association of Physicists in Medicine (AAPM) across the Southern California Chapter and Northern California Chapter. Furthermore, we have sought input from respondents regarding various strategies for mitigating job burnout and stress, ultimately striving to enhance overall work‐life balance.

## 2 METHODS AND MATERIALS

The online survey platform SurveyMonkey was used to generate a 14‐question survey to gather data from a large pool of respondents. The 14 survey questions were designed to assess respondent demographics, measure eight different variables in relation to medical physicist job status, and collect feedback regarding burnout and job stress. These eight variables are: Burnout Level, Pandemic Affected, Leaving Job, Work Environment, Salary Satisfaction, Benefit Satisfaction, Job Satisfaction Change, and Stress Level. Each variable was measured using a Likert scale ranging from 0 to 10 (see Supplementary). For example, the variable “Leaving Job” accesses whether MPs plan to leave their current job, a score of “0” means “not planning to leave at all”, 5 indicates “somewhat want to leave”, and 10 signifies “extremely wanting to leave”. The variable “Pandemic Affected” evaluates the impact of the COVID‐19 on MPs’ work and life. A score of “0” indicates “not affected at all”, 5 represents “moderately affected”, and 10 signifies “extremely affected”. The variable “Stress Level” estimates MP's job‐related stress. A score of “0” indicates “no stress”, 5 represents “somewhat stressed”, and 10 signifies “extremely stressed”. The variable “Burnout Level” estimates MP's job‐related stress. A score of “0” indicates “no burnout”, 5 represents ″somewhat burnout, and 10 signifies “extremely burnout”. Respondents were asked to assess these factors subjectively between 0–10.

The AAPM Southern California Chapter Education Committee emailed out the survey link to all AAPM Southern California (SCC) and Northern California chapter (NCC) members, requesting that each person fill out the survey only once. Our data collection period spanned 6 weeks, from January 5, 2022, to February 18, 2022. Out of a total of 338 emails sent, 97 responses were collected at the end of our collection period. All responses were anonymized, and data were analyzed using both qualitative and quantitative methods. Quantitative analysis was performed with SAS Enterprise Guide statistical software (Version 9.4).

## 3 RESULTS

At the end of the 6‐week collection period, we received a response rate of approximately 28.7%, 97 completed responses out of 338 invitations. Of the 97 responses, there were 65 respondents from Southern California and 32 from Northern California. This response rate of 28.7% is higher than that of the 2012 ASTRO comprehensive workforce study,^9^ which has a response rate of 18%.

## 4 DEMOGRAPHICS

The results indicate that among the respondents, 46% have been working at their current job for more than 10 years, 19% have been in their current position for 6 to 10 years, 29% for 1 to 5 years, and 6% for less than 1 year. This is in general agreement with the ASTRO workforce survey study.^9^ Regarding the workplace, 41% of respondents are employed at a university medical center, 27% at a community hospital, 5% are self‐employed, 16% work at a private clinic, and 10% indicated “other”. In comparison, the ASTRO survey found that the greatest number of MPs (40.8%) reported working at the hospital, 25.5% working at an academic, and 33.7% working at a private/Group practice.

## 5 STATISTICAL DATA ANALYSIS/QUANTITATIVE ANALYSIS

We collected the Likert scale responses from a total of 97 participants, with 65 from SCC and 32 from NCC. Using this data, for the eight variables, we calculated the overall mean and standard deviation for the entire group (97 participants), as well as the mean and standard deviation (Std Dev) for the 65 SCC members and the 32 NCC members. We also used an unpaired T‐test to examine if there were any significant differences between the SCC and NCC groups. All the calculated data are presented in Table 1. Based on the P values from the lowest to highest, the eight variables are ranked as follows: Leaving Job, Burnout Level, Job Satisfaction Change, Benefit Satisfaction, Pandemic Affected, Stress Level, Work Environment, and Salary Satisfaction.

**TABLE 1:** The calculated mean values of answers for Likert scale questions for the total numbers, SCC and NCC numbers, and different *p*‐values by using the unpaired *t*‐test between SCC and NCC.

Survey questions	Total Mean	Std Dev	SCC Mean	Std Dev	NCC Mean	Std Dev	p‐value
**Leaving Job**	4.94	3.77	4.35	3.61	6.07	3.89	**0.12**
**Burnout level**	5.64	2.68	5.11	2.57	6.77	2.61	**0.16**
**Job satisfaction change**	4.36	2.14	4.22	2.20	4.68	1.97	**0.32**
**Benefit satisfaction**	6.52	2.62	6.63	2.48	6.31	2.92	**0.58**
**Pandemic affected**	5.99	2.69	5.95	2.69	6.06	2.73	**0.58**
**Stress level**	6.22	2.14	6.02	1.93	6.59	2.50	**0.82**
**Work environment**	5.01	2.69	4.98	2.67	5.03	1.41	**0.89**
**Salary satisfaction**	5.57	2.76	5.72	2.65	5.25	3.04	**0.93**

Abbreviations: NCC, Northern California chapter; SCC, Southern California chapter.

Overall, no significant differences were found in any well‐being variables between the SCC and NCC subgroups, although some differences in the mean value were observed. For example, SCC participants showed a higher intention to change jobs, as reflected in the “leaving job” variable, with the mean scores of SCC and NCC being 5.37 and 4.09, respectively; however, the *p*‐value of 0.12 indicates no significant difference. The eight *p*‐values (Table 1 column 8) ranged from a minimum of 0.12, confirming that there were no significant differences across all eight variables.

Pearson correlation coefficients (CC) were also calculated across pairs of the eight job status‐related variables. Table 2 presents Pearson's correlation coefficient values in a social science study. A Pearson's CC value with magnitude in the range of 0.5 to +1.0 indicates a strong correlation, and (0.3, 0.5) indicates a moderate correlation, with the sign indicating the direction of correlation. This tier system is reported in Table 3, where yellow indicates a moderate correlation and green represents a strong correlation. The *p‐values* are less than 0.05, meaning that these numbers are statistically significant.

**TABLE 2:** Person's correlation coefficient values in social science.

Pearson's correlation coefficient (r) values in Social science	
Between +0.5 and +1.0	Strong positive correlation
Between −0.5 and −1.0	Strong negative correlation
Between +0.3 and +0.5	Moderate positive correlation
Between −0.3 and −0.5	Moderate negative correlation

**TABLE 3:** Correlation Matrix: Pearson's correlation coefficients among 8 measured variables. Yellow indicates a moderate correlation and green represents a strong correlation.

	Burnout Level	Pandemic Affected	Leaving Job	Work Environment	Salary Satisfaction	Benefit Satisfaction	Job Satisfaction Change	Stress Level
**Burnout Level**	1							
**Pandemic Affected**	0.37 *p < *0.05	1						
**Leaving Job**	0.66 *p < *0.05	0.29 *p < *0.05	1					
**Work Environment**	0.54 *p < *0.05	0.32 *p < *0.05	0.62 *p < *0.05	1				
**Salary Satisfaction**	−0.49 *p < *0.05	−0.29 *p < *0.05	−0.38 *p < *0.05	−0.29 *p < *0.05	1			
**Benefit Satisfaction**	−0.21 *p < *0.05	−0.11 P = 0.30	−0.17 *p < *0.05	−0.06 P = 0.58	0.52 *p < *0.05	1		
**Job Satisfaction Change**	−0.48 *p < *0.05	−0.15 P = 0.14	−0.51 *p < *0.05	−0.44 *p < *0.05	0.37 *p < *0.05	0.17 *p < *0.05	1	
**Stress Level**	0.70 *p < *0.05	0.33 *p < *0.05	0.54 *p < *0.05	0.48 *p < *0.05	−0.26 *p < *0.05	−0.05 *p < *0.05	−0.40 *p < *0.05	1

According to our calculated results shown in Table 3, the variable “Burnout Level” is moderately and positively correlated with “Pandemic Affected” (CC = 0.37, *p* < 0.05); “Burnout Level” is strongly and positively correlated with “Leaving Job” (CC = 0.66, *p* < 0.05), “Burnout Level” is strongly and positively correlated with “Work Environment” (CC = 0.54, *p* < 0.05), “Burnout Level” is moderately and negatively correlated with “Salary Satisfaction” (CC = −0.49, *p* < 0.05), “Burnout Level” is moderately and negatively correlated with “Job Satisfaction” (CC = −0.48, *p* < 0.05), and “Burnout Level” is strongly and positively correlated with “Stress Level” (CC = 0.70, *p* < 0.05). The variable “Pandemic Affected” is moderately and positively correlated with “Work Environment” (CC = 0.32, *p* < 0.05). The variable “Leaving Job” is strongly and positively correlated with “Work Environment” (CC = 0.62, *p* < 0.05); “Leaving Job” is moderately and negatively correlated with “Salary Satisfaction” (CC = −0.38, *p* < 0.05); “Leaving Job” is strongly and negatively correlated with “Job Satisfaction” (CC = −0.51, *p* < 0.05); “Leaving Job” is strongly and positively correlated with “Stress Level” (CC = 0.54, *p* < 0.05). The variable “Work Environment” is moderately and negatively correlated with “Job Satisfaction” (CC = −0.44, *p* < 0.05); “Work Environment” is moderately and positively correlated with “Stress Level” (CC = 0.48, *p* < 0.05). The variable “Job Satisfaction Change” is moderately and negatively correlated with “Stress Level” (CC = −0.40, *p* < 0.05).

As a fundamental principle in statistics, it is important to note that just because two variables are correlated, it does not necessarily mean that one variable is causing the other to change. Although correlation coefficients do not necessarily imply a cause‐and‐effect relationship between the two variables, these valuable calculations still provide a numerical measure of the strength and direction of the relationship between the two variables. This allows us to quantify the extent to which changes in one variable are associated with changes in another.

For example, the variable “Burnout Level” is moderately and positively correlated with “Pandemic Affected” (CC = 0.37, *p* < 0.05); “Burnout Level” is strongly and positively correlated with “Leaving Job” (CC = 0.66, *p* < 0.05), “Burnout Level” is strongly and positively correlated with “Work Environment”(CC = 0.54,*p* < 0.05), and “Burnout Level” is strongly and positively correlated with “Stress Level”(CC = 0.70,*p* < 0.05). By calculating the correlation coefficient, we can identify that these variables tend to move together. The identification of patterns is important in predicting one variable based on another. For example, employers can use this information to predict how increasing burnout levels among employees might impact worker retention rate and the possibility of these employees leaving their jobs (CC = 0.66, *p* < 0.05). We can examine the association between higher burnout levels, a higher consideration to leave one's job, a more toxic work environment, and higher levels of stress. A similar analysis can be done when observing the relationships among the other measured variables. For example, the higher an individual ranks considering leaving their job, it is correlated with a toxic work environment, lower job satisfaction, and higher stress levels.

On the other hand, “Burnout Level” is moderately and negatively correlated with “Salary Satisfaction” (CC = −0.49, *p* < 0.05); and “Burnout Level” is moderately and negatively correlated with “Job Satisfaction” (CC = −0.48, *p* < 0.05) (Table 2). By calculating the correlation coefficient, we can identify that “Burnout Level” tends to move in opposite directions with “Salary Satisfaction” and “Job Satisfaction”. This relationship can let employers know that a decrease in employee job satisfaction is associated with an increase in employee burnout levels. Similarly, a decrease in employee salary satisfaction is associated with an increase in employee burnout levels. It is important to measure and evaluate job status and burnout among medical physicists. Long‐term implications of job stress and burnout can have significant and long‐term implications for individuals and organizations. Prolonged exposure can lead to physical and mental health consequences, reduced job performance, higher rates of turnover, and contribute to a negative organizational culture.

## 6 QUALITATIVE ANALYSIS/ FREE‐TEXT RESPONSES ANALYSIS

Out of a total of 97 respondents, 64 participants chose to answer the optional open‐ended question about recommendations for improving emotional well‐being and job satisfaction. Based on the frequency of occurrence of specific keywords and themes, the seven most common recommendation themes are: adequate staffing, building a culture of recognition, salary increases, team building, structure and organization, remote work option, and physicists’ engagement in decision‐making.

From our qualitative data, we identified the most popular feedback for addressing job stress and burnout that can contribute to moving more medical physicists from the high‐strain jobs category to the active jobs category. Gameiro's study^4^ further supports that “in organizational practice, job control is a crucial characteristic to face job demands, as job control will buffer job demands’ harmful effects on workplace well‐being”. Our qualitative data feedback from respondents supports the concept that this feedback would help lower the rate of burnout and job stress, which is supported by the model. For example, one feedback from our survey is maintaining adequate staffing of medical physicists, which can alleviate overall job demand for employees. Another example from our survey is incorporating medical physicists in decision‐making. This cannot only help employees feel part of the larger team but also help increase each person's job decision latitude. Our recommendations highlight common challenges faced by medical physicists in departments throughout California related to job stress and burnout. We recognize that each department, structure, facility, and leadership operates differently; however, employers should consider both near‐and long‐term actions that mitigate burnout, spark engagement, and foster a supportive culture.

## 7 DISCUSSION

This study was set out to analyze the well‐being and job satisfaction of therapeutic medical physicists, while also gathering feedback on addressing job stress and burnout during the COVID‐19 pandemic in California. The mixed‐methods research design provided a more comprehensive understanding of medical physicists’ job status during this period. Correlation coefficients are valuable tools that provide data‐driven insights to understand and quantify relationships between eight different job status variables. Understanding contributors to job burnout is essential for the well‐being of healthcare providers, the safety of patients, the sustainability of the healthcare workforce, and the overall quality and effectiveness of healthcare services. The qualitative data provide insight into participants’ perspectives, attitudes, and experiences, complementing the quantitative findings. It also offers the seven most common recommendations for achieving a sustainable workforce and improving work‐life balance for medical physicists. Both quantitative and qualitative analyses help inform new workplace interventions and implementations, fostering a healthier and more resilient healthcare workforce. It is important for both employers and employees to acknowledge that while burnout may manifest at the individual level, its roots are deeply embedded within workplace systems and culture. In the words of U.S. former Surgeon General Vivek Murthy,^10^ the nation's health relies on the well‐being of our healthcare workforce, making the rectification of long‐standing burnout factors among healthcare professionals a top national priority.

## 8 CONCLUSION

Job stress, burnout, and workforce stability became major concerns for healthcare workers during the COVID‐19 pandemic. This study proposes a new multivariable model to assess medical physicists’ well‐being and job satisfaction in California, providing a quantitative framework for workforce modeling and future projections. Our initial study identified eight key variables and seven common recommendations to improve work‐life balance. Since this pilot study was conducted in California, its findings may not be generalizable to the broader US or global medical physics workforce. Expanding the survey to a national or global level and including other radiation oncology professionals would allow for a more comprehensive measurement and analysis of the relationships among job‐related stress, burnout, and various contributing factors. This broader approach would help develop effective strategies to alleviate job stress and ensure workforce stability in radiation oncology on a larger scale.

## AUTHOR CONTRIBUTIONS

Conception and design of the study: Xiaoyu Liu, Jennifer Zhang, Steve Goetsch. Data analysis: Fagen Xie, Jennifer Zhang. Writing and revising: Xiaoyu Liu, Jennifer Zhang, Steve Goetsch, Dan Ruan, X. Sharon Qi, Amy S. Yu, Zhilei L. Shen, and Varun Sehgal. The final manuscript was approved by all the authors. All the authors agreed to be accountable for all aspects of the work in ensuring that the questions related to the accuracy or integrity of any part of the work are appropriately investigated and resolved.

## CONFLICT OF INTEREST STATEMENT

The authors declare no conflicts of interest.

## Supporting information



Supporting Information
