# Supplementary material for: Therapeutic medical physicist well‐being and employment satisfaction during the COVID‐19 pandemic in California
Source: J Appl Clin Med Phys. 2025 Sep 1;26(9):e70233. doi: 10.1002/acm2.70233 (PMC12401932; doi:10.1002/acm2.70233)
Supplement: Supplementary file 1 — Supporting Information [file ACM2-26-e70233-s001.docx]

**Survey Questions:**

**Title: Job Status Survey- Job Stress, Burnout and Job Satisfaction**

1. **How many years have you been at your present job?**

Less than one year

1-5 years

6-10 years

More than 10 years

1. **Who is your employer?**

University Medical Center

Community Hospital

Private Clinic

Self-Employed

Other (please specify)

1. **Who do you report to?**

Department Director

Chief Physicist

Senior Physicist

Administrator

Other (please specify)

1. **How many people do you supervise?**

None

1 to 3

4 to 10

More than 10

1. **At this point in your career, what is your overall level of stress?**

**0** 1 2 3 4 **5** 6 7 8 9 **10**

0=No Stress 5=Medium Stress 10=Extremely Stress

1. **What is your level of job burnout?**

0 1 2 3 4 5 6 7 8 9 1

0=No Burnout 5=Medium Burnout 10=Extremely Burnout

1. **How much has the pandemic affected your work life?**

0 1 2 3 4 5 6 7 8 9 10

0=Not at all 5=Medium Affected 10=Extremely Affected

1. **Have you considered leaving your job?**

0 1 2 3 4 5 6 7 8 9 10

0=Not at all 5=Somewhat 10=Extremely

1. **How would you describe your work environment?**

0 1 2 3 4 5 6 7 8 9 10

0=Ideal 5=Satisfactory 10=Toxic

1. **Are you satisfied with your salary?**
2. 1 2 3 4 5 6 7 8 9 10

0=Not at all 5=Somewhat 10=Extremely

1. **Are you satisfied with your work’s benefits?**

0 1 2 3 4 5 6 7 8 9 10

0=Not at all 5=Somewhat 10=Extremely

1. **Over the last five years, how has your job satisfaction changed?**

0 1 2 3 4 5 6 7 8 9 10

0=Much worse 5=No change 10=Improve a lot

1. **If you had the opportunity to work from home during the COVID-19 pandemic, how willing are you to transition back working full-time in the office**

0 1 2 3 4 5 6 7 8 9 10

0=Not at all 5=Somewhat 10=Extremely

1. **Do you have any recommendations for your department to minimize your feelings of job-related stress and/or burnout? Please type them below:**
